# AMPK/mTOR Pathway Is Involved in Autophagy Induced by Magnesium-Incorporated TiO_2_ Surface to Promote BMSC Osteogenic Differentiation

**DOI:** 10.3390/jfb13040221

**Published:** 2022-11-05

**Authors:** Guifang Wang, Jiaxin Luo, Yuqin Qiao, Dongdong Zhang, Yulan Liu, Wenjie Zhang, Xuanyong Liu, Xinquan Jiang

**Affiliations:** 1 Department of Prosthodontics, Shanghai Ninth People’s Hospital, Shanghai Jiao Tong University School of Medicine, 639 Zhizaoju Road, Shanghai 200011, China; 2 College of Stomatology, Shanghai Jiao Tong University, National Center for Stomatology, National Clinical Research Center for Oral Diseases, Shanghai Key Laboratory of Stomatology, Shanghai Engineering Research Center of Advanced Dental Technology and Materials, 639 Zhizaoju Road, Shanghai 200011, China; 3 State Key Laboratory of High Performance Ceramics and Superfine Microstructure, Shanghai Institute of Ceramics, Chinese Academy of Sciences, No. 1295 Dingxi Road, Shanghai 200050, China

**Keywords:** magnesium, surface modification, titanium, osteogenic activity, autophagy

## Abstract

Magnesium has been extensively utilized to modify titanium implant surfaces based on its important function in promoting osteogenic differentiation. Autophagy has been proven to play a vital role in bone metabolism. Whether there is an association between autophagy and magnesium in promoting osteogenic differentiation remains unclear. In the present study, we focused on investigating the role of magnesium ions in early osteogenic activity and the underlying mechanism related to autophagy. Different concentrations of magnesium were embedded in micro-structured titanium surface layers using the micro-arc oxidation (MAO) technique. The incorporation of magnesium benefited cell adhesion, spreading, and viability; attenuated intracellular ATP concentrations and p-mTOR levels; and upregulated p-AMPK levels. This indicates the vital role of the ATP-related AMPK/mTOR signaling pathway in the autophagy process associated with osteogenic differentiation of bone marrow mesenchymal stem cells (BMSCs) induced by magnesium modification on titanium surfaces. The enhanced osteogenic differentiation and improved cellular autophagy activity of BMSCs in their extraction medium further confirmed the function of magnesium ions. The results of the present study advance our understanding of the mechanism by which magnesium regulates BMSC osteogenic differentiation through autophagy regulation. Moreover, endowing implants with the ability to activate autophagy may be a promising strategy for enhancing osseointegration in the translational medicine field in the future.

## 1. Introduction

Titanium and its alloys have been widely used as dental implants due to their excellent biocompatibility [1]. To improve the surface bioactivity and strengthen the binding ability of implants and bone tissues, various methods have been utilized for the surface modification of titanium. Magnesium is the fourth most abundant cation in the human body and the second largest cation in cells. It can be incorporated into titanium substrates using various methods to improve surface bioactivity and has been widely used to promote bone growth and regeneration. However, the role of magnesium in bone mineralization is controversial. It has been reported that appropriate concentrations of magnesium can promote the mineralization of BMSCs [2,3], while excessive magnesium can also impair their osteogenesis [4]. The entire osteogenesis process involves early osteogenic differentiation and late extracellular mineralization. During the osteogenesis process, especially the early osteogenic differentiation stage induced by magnesium ions, cell signaling triggers gene expression downstream. However, the underlying mechanisms of the facilitation effect of magnesium in regulating cell signal transduction are not fully understood.

Autophagy is an intracellular degradation process in eukaryotic cells that can transfer cytoplasmic components, including damaged macromolecules and organelles, to lysosomes for degradation and recycling [5]. It plays a very important role in the growth, mature differentiation, and homeostasis maintenance of cells and organisms [6]. Autophagy is considered to be highly involved in bone metabolism [7]. Increasing evidence suggests that an appropriate level of autophagy enables bone cells to survive hypoxic, nutrition-deficient, or even hypertonic environments. The induction of cellular autophagy plays a vital role in preosteoblast differentiation as well as osteoblast–osteocyte transitions [8,9,10]. Recent studies found that modulating magnesium transporters could accelerate osteogenic differentiation, partly via the activation of autophagy, giving clues about the correlation between magnesium, autophagy, and osteogenic differentiation [11,12]. Studies have also discovered that high concentrations of magnesium attenuated the osteogenic differentiation and mineralization ability of bone-related cells by modulating basal cellular autophagy [13,14]. Incorporating an appropriate concentration of magnesium on titanium surfaces is believed to be beneficial for improving its bioactivity as well as osteogenic differentiation. However, whether there is an association between the facilitation effect on early-stage osteogenic differentiation induced by magnesium and the modulation of autophagy remains unclear.

In the present study, we utilized the MAO technique to prepare magnesium-incorporated porous dioxide films on titanium surfaces and further investigated the role of magnesium in modulating autophagy and its possible signaling pathway in enhanced early osteogenic activity.

## 2. Materials and Methods

### 2.1. Fabrication and Modification of Specimens

Commercially pure titanium plates (Cp Ti, TA1, purity 99.85%) were ground with 400# abrasive paper and then ultrasonically washed with acetone, ethyl alcohol, and distilled water prior to plasma electrolyte oxidation (PEO) treatment. PEO was conducted in electrolytes composed of 0.1 mol/L calcium acetate monohydrate (CA, C_4_H_6_O_4_Ca·H_2_O), 0.05 mol/L glycerophosphate disodium salt pentahydrate (GP, C_3_H_7_Na_2_O_6_P·5H_2_O) and various amounts (0.01, 0.02, and 0.03 mol/L) of magnesium acetate tetrahydrate (Mg(CH_3_COO)_2_·4H_2_O) to prepare Mg-free (M0) and Mg-incorporated (M1, M2, and M3) coatings. These coatings were formed at a current density of 16.5 A/dm^2^ and frequency of 800 Hz, with a duty cycle of 10%.

### 2.2. Surface Characterization of Specimens

The surface morphology and elemental composition of Mg-free and Mg-incorporated coatings were examined and measured by scanning electron microscopy (Hitachi, Tokyo, Japan) and energy-dispersive X-ray spectrometry (EDS) (Hitachi, Tokyo, Japan) attached to an electron probe X-ray microanalysis system (JEOL, Akishima, Japan), respectively. Their phase composition was analyzed by X-ray diffraction (XRD) (Bruker, Karlsruhe, Germany). The pore size and pore distribution in three random fields of each sample were measured by ImageJ (National Institutes of Health, Bethesda, MD, USA, V1.8.0).

The surface wettability of all coatings was assessed using a contact angle instrument (Solon, Shanghai, China). For this process, 2 μL of ultrapure water was vertically dropped on the sample surface, and the contact angles of the droplets were analyzed. Three samples per group were measured, and the average was taken as the final value.

For Mg ion release, each titanium substrate (1 × 1 cm) was immersed in 10 mL deionized water for 3 days, then moved to fresh 10 mL deionized water for the next 3 days. The accumulated extract was collected, and the Mg ion concentration was detected by inductively coupled plasma atomic emission spectrometry (ICP-AES) (Agilent, Palo Alto, CA, USA).

### 2.3. Cell Culture

Four-week-old SD rats were provided by the SPF Experimental Animal Center, Ninth People’s Hospital, affiliated with the Shanghai Jiao Tong University School of Medicine. All animal protocols were approved by the Animal Care and Experiment Committee of the Ninth People’s Hospital (SH9H-2020-A619-1). BMSCs were isolated and cultured according to our previously published procedures [15]. SD rats were first anesthetized with chloral hydrate; then, both femurs were obtained. The marrow cavity was rinsed twice with high-glucose DMEM (Shanghai BasalMedia Technologies, Shanghai, China) containing 10% fetal bovine serum (Biological Industries, Kibbutz Beit Haemek, Israel). The cells were centrifuged at 1800 rpm for 10 min and subsequently cultured with 10 mL fresh high-glucose DMEM in a humidified atmosphere of 95% air and 5% CO_2_. The culture medium was changed 48 h later, and cells at passages 2–3 and 80–90% confluency were used for subsequent studies.

### 2.4. Cell Adhesion and Spreading

Cells were seeded on titanium surfaces for 24 h, then were fixed with 4% paraformaldehyde. For permeabilization, cells were treated with 0.2% Triton X-100 (Yeasen Biotechnology, Shanghai, China) in PBS for 20 min at room temperature. Then, actin microfilaments were stained with phalloidin–iFluor 488 solution (Yeasen Biotechnology, Shanghai, China) at a concentration of 100 nM for 90 min, and nuclei were stained with DAPI at a concentration of 1 μg/mL for 10 min. The number of nuclei in five random fields of each sample was counted by ImageJ to represent cell adhesion ability. The extension area of cells in three random fields of each sample was measured by ImageJ to represent cell spreading.

### 2.5. Cell Morphology and Metabolic Activity Assay

BMSCs were cultured on the substrates for 3 days. Then, the cells were fixed with 2.5% glutaraldehyde. After being dehydrated by increasing concentrations of ethanol (30, 50, 75, 90, 95, and 100%) and dried by hexamethyldisilazane, they were sputter-coated with gold and observed by SEM. The metabolic activity of rat BMSCs was determined by MTT assay. After 4 and 7 days of culture, the cell metabolic activity was measured by comparing the optical density (OD) at 540 nm with the absorbance at 630 nm. The MTT assay was performed in triplicate, and each sample was analyzed twice.

### 2.6. Live/Dead Assay

BMSCs were cultured on substrates for 3 days; then, a live/dead staining assay was conducted using a Calcein–AM/PI double staining kit according to the manufacturer’s protocol (Yeasen Biotechnology, Shanghai, China). Briefly, cells were washed with assay buffer three times; then, calcein AM (2 μM) and propidium iodide (1.5 mM) solutions were added to each well, and cells were incubated at 37 °C for another 30 min. The specimens were subsequently observed under a confocal microscope (Leica, Wetzlar, Germany).

### 2.7. Luciferase Assay

ATP concentration was measured by using a luciferase assay according to the manufacturer’s protocol (Beyotime Biotechnology, Shanghai, China). After cells were cultured on titanium surfaces for 2 and 4 days, they were lysed and centrifuged at 12,000 rpm for 10 min. The collected supernatant was mixed with ATP detection solution; then, the RLU value was determined by a luminometer (Tecan, Männedorf, Switzerland). The ATP concentration was calculated according to the standard ATP concentration curve. A Micro BCA protein assay kit (Thermo Fisher Scientific, Waltham, MA, USA) was used to detect the concentration of total protein. The ratio of ATP to total protein (mg) represented the relative intracellular ATP level. The luciferase assay was performed in triplicate, and each sample was analyzed twice.

### 2.8. Reverse-Transcription Real-Time Polymerase Chain Reaction Assay

Cells were cultured on substrates to examine the direct effects of magnesium. To determine the effect of dissolved magnesium ions on cell bioactivity, extracts were collected using the following procedure. Briefly, titanium samples were immersed in 1 mL DMEM for 3 days, then moved to fresh 1 mL DMEM for another 3 days. Then, the collected extracts were supplemented with 10% fetal bovine serum. After 7 days of culture on substrates or in extracts, total RNA was extracted using TRIzol reagent. Complementary DNA was synthesized by using a PrimeScript RT reagent kit. The primer sequences were as follows: ALP: forward: GTCCCACAAGACCCCACAAT, reverse: CAACGGCAGAGCCAGGAAT; OCN: forward: GCCCTGACTGCATTCTGCCTCT, reverse: TCACCACCTTACTGCCCTCCTG; Runx2: forward: ATCCAGCCACCTTCACTTACACC, reverse: GGGACCATTGGGAACTGATAG; BMP2: forward: TGAACACAGCTGGTCTCAGG, reverse: TGACGCTTTTCTCGTTTGTG. The expression of these genes was quantified by real-time polymerase chain reaction (PCR) with SYBR Premix Ex TaqII (TaKaRa, Osaka, Japan). The relative expression levels for these genes were normalized to that of the housekeeping gene, GAPDH (forward: GGCAAGTTCAACGGCACAGT, reverse: GCCAGTAGACTCCACGACAT). The real-time PCR assay was performed twice, and each sample was analyzed in triplicate.

### 2.9. Immunofluorescence Microscopy

After 4 days of incubation, the expression of p-mTOR, p-AMPK, OCN, and LC3B of BMSCs cultured on titanium surfaces was identified by polyclonal rabbit antibodies (all from Beyotime Biotechnology, Shanghai, China) against p-mTOR (1:200), p-AMPK (1:200), OCN (1:200), and LC3B (1:200). Alexa Fluor 594 AffiniPure donkey anti-rabbit IgG (1:200; Yeasen Biotechnology, Shanghai, China) was further conjugated on the primary antibody. Actin microfilaments were stained by phalloidin–iFluor 488 solution (Yeasen Biotechnology, Shanghai, China). Nuclei were stained by DAPI solution (Beyotime Biotechnology, Shanghai, China) for 10 min. The samples were subsequently photographed by a confocal microscope (Leica, Germany).

### 2.10. Adenovirus Transfection

Cells were first seeded on the titanium surfaces to reach a confluency of about 70–80%; then, adenoviral mCherry-GFP-LC3B (Beyotime Biotechnology, Shanghai, China) with a multiplicity of infection (MOI) ratio of 20 was used for transfection for 24 h. Subsequently, cells were placed in a fresh culture medium and cultured for another 24 h for transfection confirmation. After another day of culture, specimens were fixed with 4% paraformaldehyde, and the fluorescence expression was observed under a confocal microscope (Leica, Germany).

### 2.11. Western Blotting Analysis

The Western blotting analysis was performed using standard techniques. Cells were cultured in different extracts for 3 days; then, protein samples were collected. Equal amounts of protein were placed on SDS-PAGE gel for electrophoretic separation. Proteins were then transferred to a pre-activated PVDF membrane. The membrane was incubated with polyclonal rabbit antibodies against OCN (1:1000), LC3B (1:1000), p-AMPK (1:1000), and p-mTOR (1:500) and monoclonal mouse antibody against β-actin (Wuhan Servicebio Technology, Wuhan, China) at 4 °C overnight, and then incubated with goat anti-rabbit peroxidase-conjugated secondary antibodies (Yeasen Biotechnology, Shanghai, China) at room temperature for 1 h. The protein band was scanned by the UVItec ALLIANCE 4.7 gel imaging system. The grey value of the typical protein band was calculated by ImageJ. The band intensity of each targeted protein normalized to β-actin represented its relative expression.

### 2.12. Statistical Analysis

The experimental data, expressed as mean ± standard deviation, were analyzed by SPSS 19.0 statistical analysis software (SPSS, Chicago, IL, USA). Statistical differences (*p* < 0.05) among various groups were compared using one-way ANOVA and SNK analysis.

## 3. Results

### 3.1. Surface Characterization

**Figure 1:**
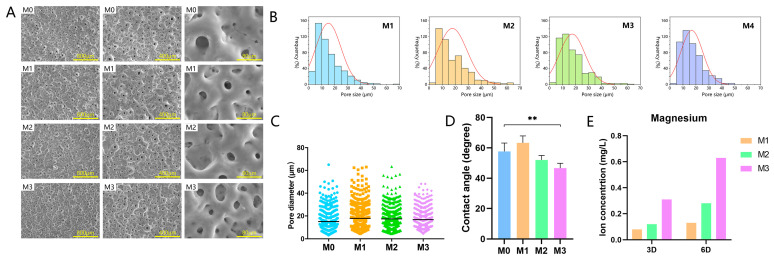
(**A**) Surface morphology observation of four samples at different magnifications. (**B**) Frequency distribution of pore size. (**C**) Pore size and (**D**) wettability of four substrates. (**E**) Accumulated Mg ion release from different specimens (** *p* < 0.01).

Figure 1A shows the surface morphology of dioxide titanium surfaces. After MAO treatment, a typical micro-porous network with a crater-like structure was observed on sample M0. The surface morphology seemed to have no obvious change after magnesium was doped into the coating. Figure 1B,C shows the pore distribution and average pore size of the four groups, with pores of around 3–70 μm and an average pore size of 15–20 μm. Most of the pores ranged in size from 5 to 25 μm. There were no significant differences in pore distribution and pore size among the four groups. The wettability of these samples was examined by water contact angle analysis. The contact angles were 57.62 ± 4.83, 63.27 ± 4.01, 52.05 ± 2.50, and 46.66 ± 2.74 for sample groups M0, M1, M2, and M3, respectively (Figure 1D). These four groups all showed a certain surface hydrophilicity, while the contact angle was slightly decreased in M3. The detection of magnesium ion release from these coatings is illustrated in Figure 1E. Magnesium ions were successfully detected in the extracts of M1, M2, and M3 at low concentrations ranging from 0.08 to 0.63 mg/L. The magnesium ion concentration increased with increased magnesium content on the titanium surface.

**Figure 2:**
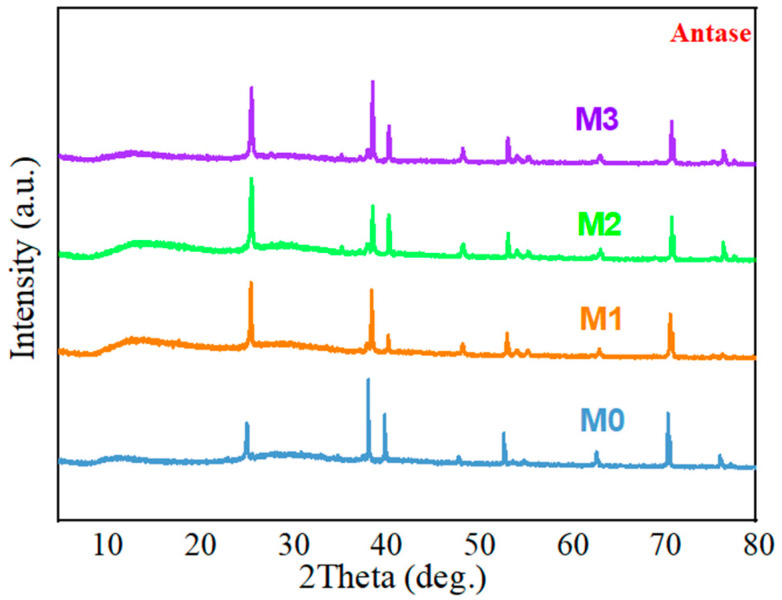
XRD patterns of four titanium substrates.

X-ray diffraction (XRD) analysis was performed on various samples to determine the crystallinity of the titanium dioxide layer. As shown in Figure 2, typical peaks of titanium and anatase TiO_2_ phase were detected on the four titanium surfaces. In general, there was no significant alteration in surface morphology, wettability, or phase composition of these coatings after magnesium was incorporated.

Dispersive X-ray detector (EDX) analysis was performed to examine the chemical composition of titanium surfaces. As illustrated in Table 1, oxygen and titanium were the main elements on the four titanium substrates. Both Pi and Ca were detected in the four groups, with small differences among them. No magnesium was detected in group M0, while it was detected in the magnesium-containing electrolytic treatment groups. The magnesium percentage was 0.87 ± 0.16, 1.72 ± 0.04, and 2.51 ± 0.04% for groups M1, M2, and M3, respectively.

### 3.2. Bioactivity and Biocompatibility

**Figure 3:**
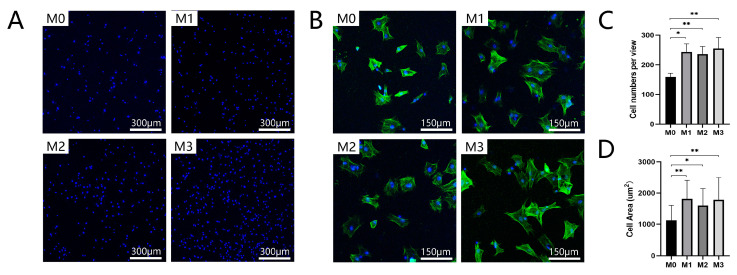
(**A**,**C**) Cell adhesion and (**B**,**D**) cell spreading assays were performed after BMSCs were cultured on coatings for 24 h (** *p* < 0.01, * *p* < 0.05).

The results of cell adhesion and spreading are shown in Figure 3A,B. After cells were incubated on the samples for 24 h, more attached cells were observed in the magnesium-incorporated groups than in the control group. The statistical analysis showed that there were no significant differences among the magnesium-incorporated groups. As shown in Figure 3C,D, the coverage area of attached cells was significantly larger in groups M1, M2, and M3 than in the group M0. There were no significant differences among groups M1, M2, and M3.

**Figure 4:**
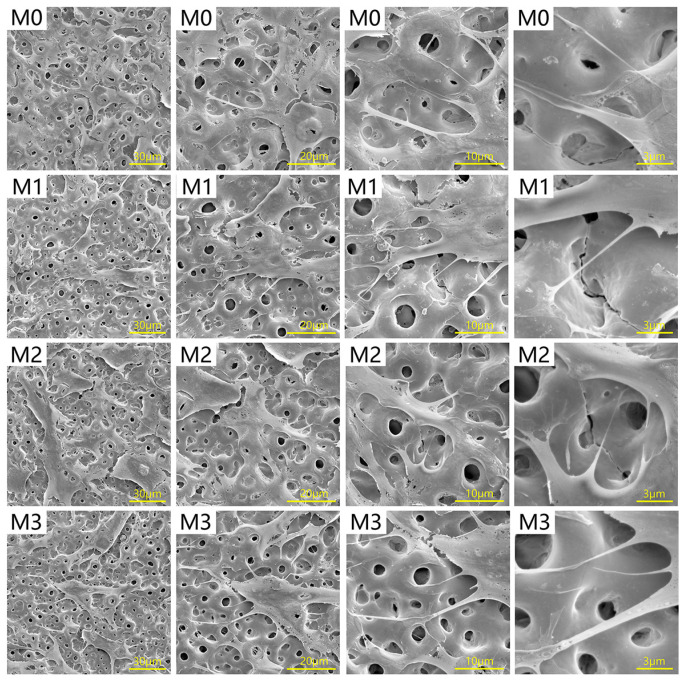
SEM observation of morphology of cells cultured on Mg-free and Mg-incorporated coatings for 3 days at different magnifications.

The results of cell morphology observation are shown in Figure 4. After 3 days of culture, cells were observed to grow well on the four titanium specimens. There were numerous pseudopodia extending from the cell surface attached to the micro-porous titanium surfaces.

**Figure 5:**
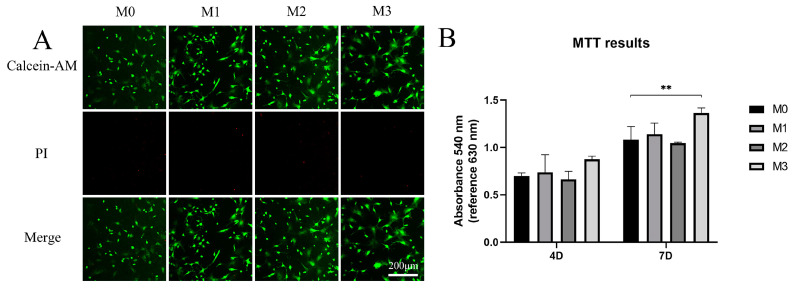
(**A**) Live/dead assay of cells cultured on titanium surfaces for 3 days. (**B**) MTT assay of cell viability (** *p* < 0.01).

The live/dead assay results are presented in Figure 5A. The majority of cells were alive, and few dead cells were observed on the titanium surfaces, suggesting good biocompatibility for cell growth. The results of the MTT assay, shown in Figure 5B, represent total cell metabolic activity. After 4 days of culture, no significant differences were observed between Mg-free and Mg-incorporated titanium oxide surfaces. After 7 days of culture, total cell metabolic activity was higher on the M3 titanium surface than on the other three (M0, M1, and M2). The addition of magnesium showed good biocompatibility for cell viability, while a high magnesium content improved cell metabolic activity.

### 3.3. Osteogenic Differentiation

**Figure 6:**
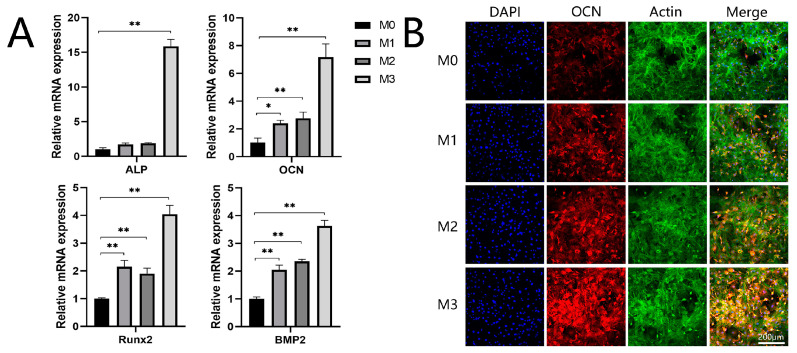
(**A**) Expression of osteogenic-related ALP, OCN, Runx2, and BMP2 genes. (**B**) Immunofluorescence staining for OCN protein (** *p* < 0.01, * *p* < 0.05).

Cells were cultured on different coatings for 7 days. A real-time PCR assay was performed to examine the mRNA expression of osteogenic-related genes, including ALP, OCN, Runx2, and BMP2. As illustrated in Figure 6A, the expression of ALP was upregulated in the three magnesium-incorporated groups, while the mRNA expression of ALP on the M3 coating was significantly higher compared to the other groups. The expressions of OCN, Runx2, and BMP2 were significantly improved on the Mg-incorporated coatings compared to the control group. M3 exhibited the highest expression of ALP, OCN, Runx2, and BMP2 mRNA among the four groups. As shown in Figure 6B, the intensity of immunofluorescence staining of OCN protein was the lowest in the control group and was more pronounced in the three magnesium-incorporated groups. Among the four groups, M3 exhibited the strongest OCN protein expression.

### 3.4. Autophagy Activation and Its Possible Mechanism

**Figure 7:**
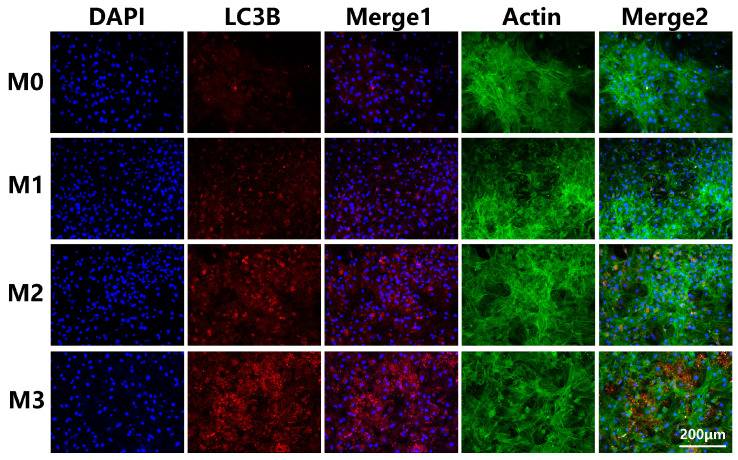
Immunofluorescence staining for LC3 protein.

Previous investigations demonstrated that autophagy plays an important role in the process of osteogenic differentiation of stem cells. In this study, we examined the protein level of autophagic marker LC3 to assess cellular autophagic activity. After 4 days of culture, the LC3 protein expressions of BMSCs cultured on different titanium surfaces were determined by immunofluorescence staining; and a typical image is shown in Figure 7. The distribution of LC3 protein was dispersed in group M0, and only a few LC3 protein puncta could be observed, while the Mg-incorporated titanium surfaces induced LC3 protein expression and accumulation. Group M3 exhibited the strongest fluorescent intensity of LC3 protein, and its distribution was spot-like.

**Figure 8:**
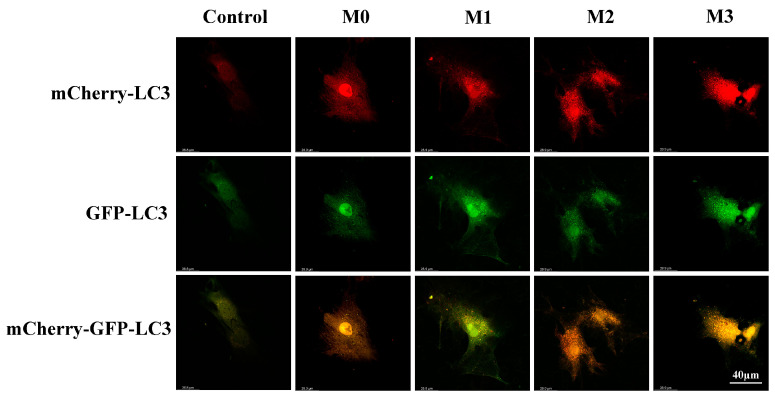
Observation of autophagy flux in cells transfected with mCherry-GFP-LC3B adenovirus.

To further elucidate the effects of incorporated magnesium on the autophagy flux of cells cultured on titanium dioxide surfaces, BMSCs were transfected with mCherry-GFP-LC3B adenovirus. Typical images in Figure 8 show that co-localization of mCherry-LC3B and GFP-LC3B dots was higher in groups M1, M2, and M3 compared to M0, indicating a higher level of autophagy. The M3 coating showed the strongest co-localization of mCherry-LC3B and GFP-LC3B dots.

**Figure 9:**
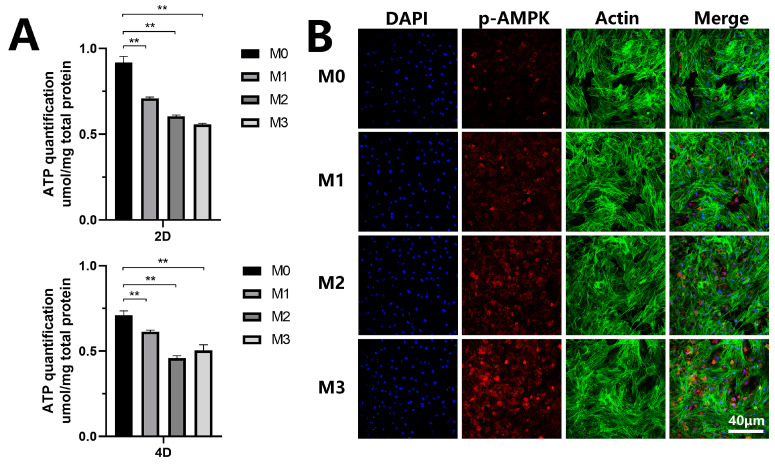
(**A**) Intracellular ATP level. (**B**) Immunofluorescence staining for p-AMPK protein (** *p* < 0.01).

In the regulation of osteogenic differentiation and autophagy, evidence has shown a critical role for the AMPK axis. AMPK senses low energy levels in cells. The sudden fall of cellular energy is known to activate AMPK, resulting in the phosphorylation of downstream targets and the inhibition of mTOR, which can result in the regulation of autophagy. To determine the potential mechanism of autophagy induction, we first examined the intracellular level of ATP using a luciferase assay. The statistical analysis is shown in Figure 9A. After 2 and 4 days of incubation, the intracellular ATP levels of cells cultured on magnesium-incorporated coatings were decreased compared to the control group. The level of p-AMPK expression was further investigated by immunofluorescence staining. As shown in Figure 9B, phosphorylation of AMPK protein was found to be strengthened in groups M1, M2, and M3 compared to the control. M3 exhibited the highest p-AMPK protein expression level.

**Figure 10:**
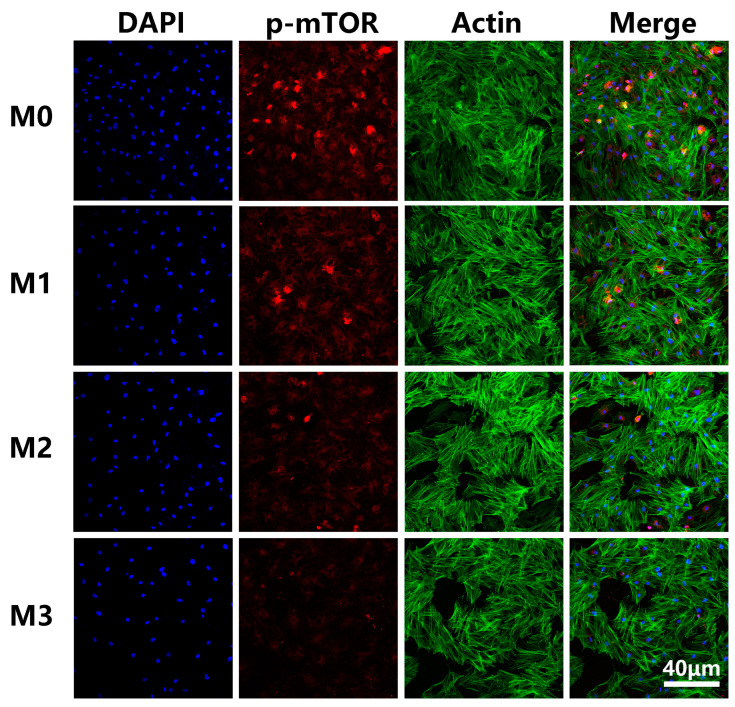
Immunofluorescence staining for p-mTOR protein.

The detection of the p-mTOR protein is shown in Figure 10. The fluorescence intensity of p-mTOR protein was obviously less pronounced in groups M1, M2, and M3 compared to M0. The M3 coating showed the lowest p-mTOR protein expression among the four specimens. The incorporated magnesium downregulated cellular energy levels in the process of enhancing osteogenic differentiation, then activated AMPK and inhibited mTOR, subsequently initiating autophagy. Taken together, these data point out that the AMPK/mTOR signaling pathway plays a vital role in autophagy-induced osteogenic differentiation regulated by magnesium.

### 3.5. The Effect of Extracts on Osteogenic Differentiation and Autophagy

**Figure 11:**
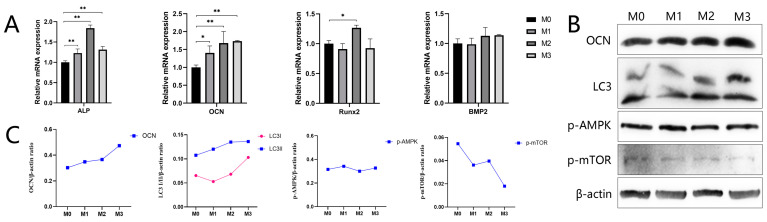
(**A**) Expression of osteogenic-related genes, ALP, OCN, Runx2, and BMP2. (**B**) Western blotting for OCN- and autophagy-related proteins. (**C**) The gay value ratio of OCN- and autophagy-related proteins to β-actin (** *p* < 0.01, * *p* < 0.05).

BMSCs were cultured in extracts of different coatings for 7 days. The results of relative mRNA expression of osteogenic-related genes (ALP, OCN, Runx2, and BMP2) are shown in Figure 11A. ALP expression was significantly upregulated in the three magnesium-containing titanium extracts compared to the M0 group, while M2 exhibited the highest expression level of ALP mRNA. OCN expression was significantly increased in groups M1, M2, and M3 compared to the control group, and the highest level was observed in M3. Runx2 enhancement was observed in group M2, while there was no significant change observed in M1 and M3 compared to the control group. The expression of BMP2 was slightly improved in M2 and M3 compared to M0, although the difference was not significant. After cells were incubated in different extracts for 4 days, the protein expression of OCN, LC3, p-AMPK, and p-mTOR was examined by Western blotting analysis (Figure 11B,C). OCN and LC3 II protein expression was higher in the magnesium-incorporating groups, M1, M2, and M3. M3 exhibited the deepest gray band and the highest gray value ratio among the four groups. The expression of p-mTOR showed an opposite trend to OCN and LC3 II. The expression of LC3 I was downregulated in M1 and upregulated in M2 and M3, compared to M0. The gray band of p-AMPK seemed deeper in M1 compared to the control group, and the gray value ratio of p-AMPK to β-actin was slightly higher in M1 compared to the control. The gray value ratio of p-AMPK to β-actin in M2 and M3 seemed to have no obvious enhancement.

## 4. Discussion

Magnesium is an essential inorganic component in bone tissue that plays a crucial role in skeletal development [16]. Mg deficiency has been linked to atherosclerosis, endothelial dysfunction, inflammation, insulin resistance, and dyslipidemia [17,18,19], while excess magnesium impairs the mineralization of BMSCs and thus has adverse biological effects on bone [20,21]. Therefore, local delivery of magnesium at appropriate concentrations might be a better approach for clinical application. MAO has been widely used to fabricate micro-structured ceramic oxide layers composed of several kinds of bioactive metal ions on titanium substrates [22]. In the present study, we fabricated a magnesium-containing dioxide titanium surface via the MAO technique and focused on its influence on BMSC osteogenic differentiation and its possible underlying mechanism related to autophagy regulation.

The surface topography, chemical composition, and wettability of titanium have been reported to interactively regulate and control local cytokine production and osteogenic differentiation, thus finally determining in vivo bone-implant contact. By keeping the electrolytic parameters consistent, the surface morphology, including average pore size and pore distribution, and surface crystallization remained the same among the four groups. During the MAO process, magnesium ions participated in the plasma–chemical reaction occurring at the metal–electrolyte interface and then were embedded in the porous coating formed on the titanium substrates. By adjusting the magnesium ion concentration in the electrolyte, different amounts of magnesium were embedded in the coatings. The increasing concentration of magnesium released from different coatings further confirmed that magnesium was incorporated into the surface layer of the material and thus could be released in a content-dependent manner. A small amount of incorporated magnesium ion did not change the surface wettability, while a larger magnesium content improved surface hydrophilicity, probably owing to the cumulative –OH groups on the surface of the crystals [23]. Although increased surface wettability has been well recognized for its vital function in promoting cell response, a score of degrees is usually considered as little difference. In a comparison study, an appreciable improvement in hydrophilicity from ~108° to 47° did not affect the initial single-cell detachment force and process of detachment at the titanium implant surfaces in vitro [24]. In another study, compared to a contact angle of ~65°, a contact angle of about 27° showed no obvious stimulation effect on cell integrin expression [25]. Moreover, magnesium incorporated in the scaffold was proved to be able to induce desirable protein adsorption and cell membrane integrin receptor expression, thus enhancing initial cell adhesion [26]. Based on previous studies and our data, we believe that the major difference among the four coatings is the different amounts of magnesium incorporated.

The results of ALP, OCN, Runx2, and BMP2 gene expression and OCN protein expression demonstrated the increased osteogenic differentiation ability of the magnesium-modified coatings. The higher the magnesium content, the better the enhancement effect that could be observed. The evidence described above indicates the critical role of magnesium in cell responses, including cell compatibility, adhesion, attachment and early-stage osteogenic differentiation, which suggests a potentially beneficial role in improving titanium-bone bonding.

Autophagy is an evolutionarily conserved cellular pathway that plays a crucial role in bone remodeling [27] by mediating cellular metabolism. MSCs with a high level of autophagy have increased osteogenic differentiation potential [28]. Recently, titanium surfaces with autophagy-modulating ability have attracted attention in orthopedic and dental implant research. Titanium-based rough surfaces were confirmed to promote osteogenic differentiation via the autophagy-dependent PI3/Akt signal transduction pathway [29]. Nanotopography on titanium was recognized as promoting osteogenic differentiation via autophagy-mediated signaling between YAP and β-catenin [30]. A combination of micro-nano topography exhibited an ability to create an anti-inflammation microenvironment, thus promoting cell proliferation and osteogenic differentiation by inducing cellular autophagy activity [31]. Beyond the surface topography, the chemical composition was also utilized to modulate autophagy activity on titanium surfaces in order to obtain better osteogenic differentiation [32,33]. Autophagy is greatly influenced by magnesium, but the effect is quite complex. Studies have reported that dietary magnesium deficiency reduces the number of autolysosomes and autophagosomes [34]. Magnesium incorporated in biomaterials triggers autophagy to mediate apoptosis in osteosarcoma cells [35,36]. Other studies have reported that magnesium negatively regulates cellular autophagy levels, resulting in an inhibitory effect of extracellular matrix calcification and osteogenic differentiation of ADTC cells, as well as human BMSCs [13,14,37]. This phenomenon may be explained by the fact that magnesium plays a dual role in bone metabolism while it changes the crystalline morphology of HA and inhibits collagen calcification [38]. Consistent with numerous previous studies [26,39,40], the present study demonstrated that an appropriate amount of magnesium incorporation was beneficial for BMSC proliferation as well as early-stage osteogenic differentiation. The upregulated expression of LC3 protein and enhanced autophagy influx indicated that cellular autophagy was activated in the process of osteogenic differentiation induced by magnesium incorporation. 

For a better understanding of the role of magnesium in BMSC osteogenic differentiation and autophagy activation, extracts collected from different titanium surfaces were prepared and utilized in cell cultures. Along with improved magnesium content on the titanium surface, the magnesium concentration in the extracts was increased. Its promotional effect on osteogenic differentiation and activation of cellular autophagy and its possible signaling pathway was further confirmed, suggesting a positive role of dissolved magnesium in the local environment. However, the beneficial effect was obviously attenuated when cells were cultured in the extractions, indicating that the major stimulation effect in autophagy-induced osteogenic differentiation may be attributed to the magnesium present on the titanium dioxide surface.

An exploration of the observed effects on autophagy, osteogenic differentiation, and magnesium stimulated a further investigation of the possible mechanism. Evidence has shown that there is a critical role of the AMPK axis in regulating osteogenic differentiation and autophagy. AMPK has been reported to control the osteogenic differentiation of human MSCs through early mTOR inhibition-mediated autophagy [41,42]. The AMPK activator has been demonstrated to have a role in inducing osteogenic differentiation and mineralization of osteoblastic cell lines and bone marrow progenitor cells [43,44,45,46]. A recent study reported that magnesium released from biomaterials could activate the AMPK/mTOR signaling pathway, thus upregulating autophagy in osteosarcoma cells [35]. We further explored the level of AMPK phosphorylation. Though applied to different cell lines and in different amounts, it was found that magnesium could upregulate the level of p-AMPK in a dose-dependent manner. In addition, phosphorylation levels of molecular mTOR were altered in an opposite trend to AMPK variation. Known as an energy sensor, AMPK is an evolutionarily conserved serine/threonine protein kinase that is precisely regulated by the ratios of AMP/ATP and ADP/ATP in the cells. In our study, we detected intracellular ATP levels and found that the incorporation of magnesium in the titanium dioxide surface resulted in a significant reduction in ATP levels. The cellular contents of ADP and AMP increase in response to energy stress. An increase in the intracellular AMP to ATP ratio, as a consequence of decreased intracellular ATP, may be responsible for the activation of AMPK, as well as the subsequent induction of cellular autophagy. Serving as a cofactor of ATP, magnesium plays an important role in regulating glucose metabolism [47], resulting in a metabolic shift from oxidative phosphorylation to glycolysis [48]. Compared to oxidative phosphorylation, glycolysis gives rise to faster but lower ATP production [49], which is consistent with our results showing that there was a significantly decreased amount of ATP with the presence of magnesium. In conclusion, the ATP-related AMPK/mTOR pathway is a pivotal regulator involved in early-stage osteogenic differentiation and autophagy induced by magnesium incorporation.

## 5. Conclusions

Using the MAO technique, we fabricated micro-structured titanium dioxide coatings with different contents of magnesium. The incorporation of magnesium did not obviously change the surface morphology, porosity, or surface wettability. We found that incorporating magnesium benefited cellular responses, including cell adhesion, spreading, and viability. Osteogenic differentiation and autophagy activity were more pronounced on titanium oxide coatings incorporated with magnesium. Consistently, intracellular ATP and p-mTOR levels were decreased, whereas p-AMPK levels were upregulated after magnesium modification. The findings of the present study demonstrate that the AMPK/mTOR signaling pathway is involved in the process of autophagy associated with the osteogenic differentiation of BMSCs induced by magnesium incorporation. These results advance our understanding of the link between autophagy induction and osteogenic differentiation regulated by magnesium-modified biomaterials and reveal a potential mechanism of magnesium-mediated BMSC osteogenic differentiation and autophagy.

## Figures and Tables

**Table 1:** Chemical composition of Mg-free and Mg-containing coatings detected by energy-dispersive X-ray spectra (EDX).

Groups	Element (wt.%)				
	O	P	Ca	Ti	Mg
M0	47.81 ± 0.06	11.99 ± 0.19	9.50 ± 0.31	30.71 ± 0.12	——
M1	48.30 ± 0.27	11.15 ± 0.08	7.27 ± 0.21	32.41 ± 0.34	0.87 ± 0.16
M2	48.51 ± 0.50	9.81 ± 0.18	8.35 ± 0.49	31.61 ± 0.13	1.72 ± 0.04
M3	47.61 ± 0.69	9.45 ± 0.34	7.57 ± 0.32	32.86 ± 0.21	2.51 ± 0.04

## Data Availability

Not applicable.
